# Computational analysis identifies putative prognostic biomarkers of pathological scarring in skin wounds

**DOI:** 10.1186/s12967-018-1406-x

**Published:** 2018-02-20

**Authors:** Sridevi Nagaraja, Lin Chen, Luisa A. DiPietro, Jaques Reifman, Alexander Y. Mitrophanov

**Affiliations:** 1 Department of Defense Biotechnology High Performance Computing Software Applications Institute, Telemedicine and Advanced Technology Research Center, US Army Medical Research and Materiel Command, MCMR-TT, 504 Scott Street, Ft. Detrick, MD 21702 USA; 2 Center for Wound Healing and Tissue Regeneration, College of Dentistry, University of Illinois, Chicago, 801 S. Paulina Street, Chicago, IL 60612 USA

**Keywords:** Pathological scarring, Computational modeling, Biomarkers, Predictive analysis

## Abstract

**Background:**

Pathological scarring in wounds is a prevalent clinical outcome with limited prognostic options. The objective of this study was to investigate whether cellular signaling proteins could be used as prognostic biomarkers of pathological scarring in traumatic skin wounds.

**Methods:**

We used our previously developed and validated computational model of injury-initiated wound healing to simulate the time courses for platelets, 6 cell types, and 21 proteins involved in the inflammatory and proliferative phases of wound healing. Next, we analysed thousands of simulated wound-healing scenarios to identify those that resulted in pathological (i.e., excessive) scarring. Then, we identified candidate proteins that were elevated (or decreased) at the early stages of wound healing in those simulations and could therefore serve as predictive biomarkers of pathological scarring outcomes. Finally, we performed logistic regression analysis and calculated the area under the receiver operating characteristic curve to quantitatively assess the predictive accuracy of the model-identified putative biomarkers.

**Results:**

We identified three proteins (interleukin-10, tissue inhibitor of matrix metalloproteinase-1, and fibronectin) whose levels were elevated in pathological scars as early as 2 weeks post-wounding and could predict a pathological scarring outcome occurring 40 days after wounding with 80% accuracy.

**Conclusion:**

Our method for predicting putative prognostic wound-outcome biomarkers may serve as an effective means to guide the identification of proteins predictive of pathological scarring.

**Electronic supplementary material:**

The online version of this article (10.1186/s12967-018-1406-x) contains supplementary material, which is available to authorized users.

## Background

Cutaneous hypertrophic scars (HTSs) are a common form of pathological scarring that occurs after traumatic skin injuries and surgical procedures with 17–67% incidence rate [1–3]. The resultant disfigurement, pruritus, pain, contractures, and morbidity are often very detrimental to a patient’s well-being. The pathogenesis of HTSs involves both cellular and extracellular matrix (ECM) components of the skin, which are regulated by a wide variety of proteins released at the wound site during the inflammatory, proliferative, and remodeling phases of wound healing [2, 4].

Recent experimental studies have provided a great deal of information about the pathogenesis of HTSs. Genomic, protein, and histologic differences have been identified and compared between normotrophic scars and HTSs [5–7]. HTSs are characterized by excess collagen and increased angiogenesis, as well as by the increased presence of proliferative cells and the proteins they release, such as fibronectin, transforming growth factor-β (TGF-β), and matrix metalloproteinase-9 (MMP-9) [4–6, 8, 9]. Although these characteristics are useful in understanding the pathogenesis of HTSs, informative prognostic biomarkers that can reliably predict the risk of pathological scar development are few [4]. Existing predictive indicators of HTSs include both empirical factors (e.g., appearance, size, and closure rate of a wound) and serum biomarkers [e.g., serum concentrations of TGF-β, decorin, interleukin (IL)-6, IL-10, and IL-β] [4, 10, 11]. Such predictors are rarely used clinically however, due to the lack of readily available laboratory tests to measure those proteins. The identification of reliable and readily measurable prognostic biomarkers of pathological scarring would allow for the proactive treatment of patients who are likely to develop HTSs, greatly improving clinical care. Several practical limitations have hindered the identification of prognostic biomarkers for HTS formation. Clinical studies of pathological scarring conditions, such as HTSs and keloids, often employ small sample sizes and are most frequently conducted after the onset of pathological scarring. Given these limitations, one complementary approach that allows systematic identification of promising biomarkers of pathological wound scarring is computational modeling.

We have previously developed a computational model of wound healing whose parameters were derived from in vitro cell culture experiments and validated using in vivo data from a variety of animal wound models, as well as from human wounds [12, 13]. Here, we use that model to perform predictive analysis of HTS formation. The contributions of the current study are threefold. First, we developed a computational approach to represent natural variability in normal healing and pathological scarring. Second, we developed a strategy for putative HTS biomarker discovery using model-simulated wound-healing scenarios. This strategy was validated using available in vivo data on wound healing in human subjects. Third, we performed a computational study to identify promising prognostic protein biomarkers for HTSs. Our analysis suggested that IL-10, tissue inhibitor of matrix metalloproteinase (TIMP)-1, and fibronectin levels on days 14 and 21 post-wounding could predict, with accuracies of 80 and 86%, respectively, a pathological scarring outcome occurring 40 days after wounding. The results demonstrate the power of computational modeling in identifying candidate prognostic markers for HTSs and other wound healing pathologies.

## Methods

### Computational kinetic model of wound healing

**Fig. 1:**
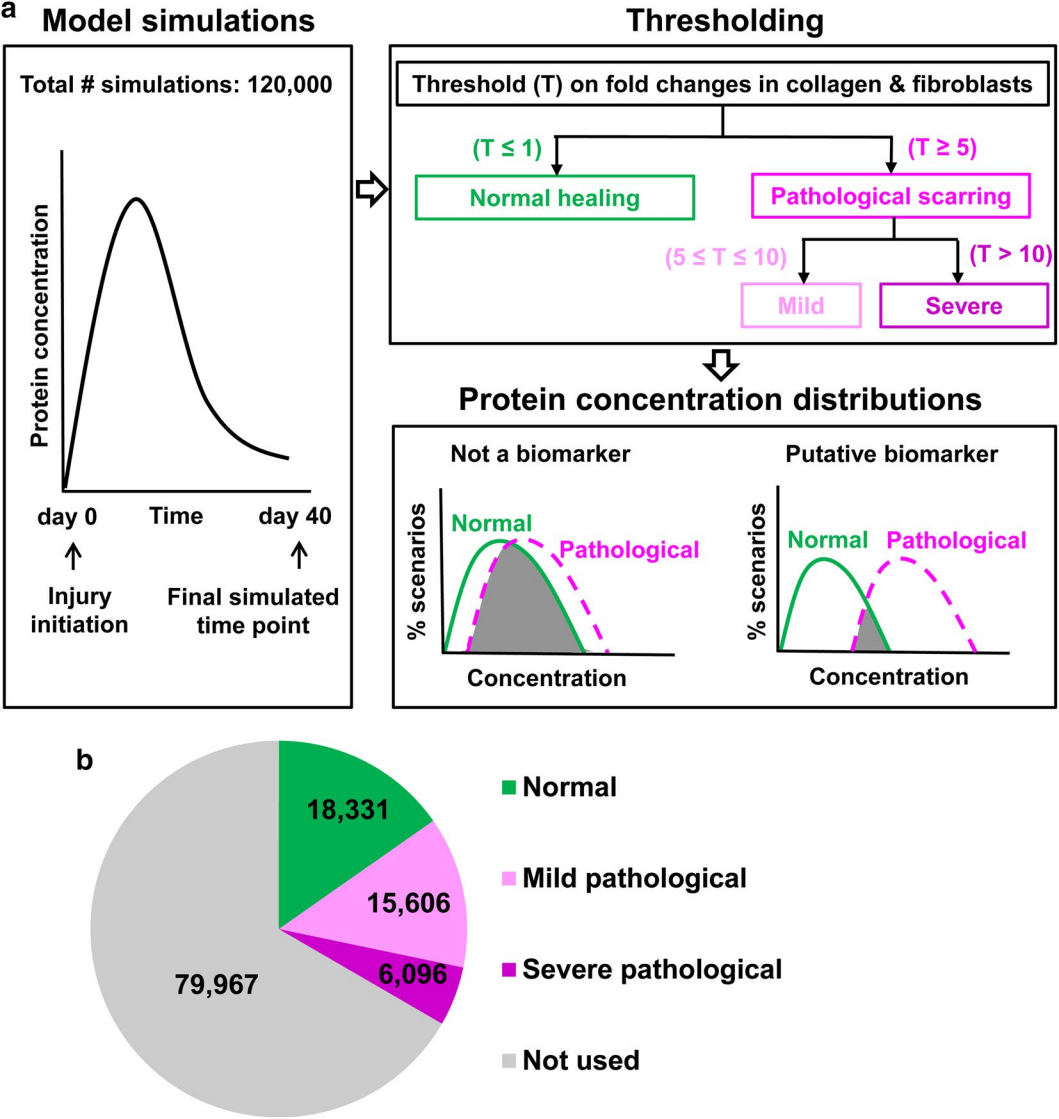
Computational strategy. **a** First, we used our computational kinetic model to simulate 120,000 distinct wound-healing scenarios. The output of each simulation comprised the time courses for the 28 model variables at 40 time points after wounding (each simulated time point represented the level of a variable on each of the 40 days post-wounding). In addition to the 120,000 simulations, we used the default parameter set to simulate a normal wound healing (i.e., normal healing) scenario. Second, we calculated fold changes (on day 40) of total collagen and fibroblast concentrations in each of the 120,000 simulations with respect to their corresponding values in the normal-healing simulation. Based on these fold changes, we classified the 120,000 simulations as “normal” (fold change ≤ 1), “mild pathological” (5 ≤ fold change ≤ 10), or “severe pathological” (fold change > 10) scarring simulations. Finally, we analysed the concentration distributions (or histograms) of 18 modeled wound proteins (excluding collagen) in the normal-healing and pathological-scarring simulations to determine the diagnostic and prognostic biomarkers of pathological scarring (see the “Methods” section for further details). **b** The pie chart shows the number of simulations that fell into each of the three categories of wound healing (i.e., normal, mild pathological, or severe pathological) after implementation of the classification criteria

We previously developed a computational kinetic model that simulated the concentration time courses for platelets, 6 cell types and 21 wound proteins (including three forms of collagen) over 40 days during an injury-initiated wound-healing response (Fig. 1) [12, 13]. A detailed list of the modeled cell types and proteins is provided in the Additional file 1. This kinetic model is system of 28 differential equations with 108 parameters. The model parameters (i.e., protein production and degradation rates, cell chemotaxis rates, etc.) were derived from in vitro cell culture data on different mammalian species including humans. In those studies, we validated the model using in vivo wound data (that had not been used in model development) from mice, pigs, rats, and humans [12, 13]. The default model parameter set corresponded to a normal wound-healing scenario (i.e., a wound that healed in a timely manner without excessive scarring). The model parameter values, parameter descriptions, model equations, and validation data are detailed in our previous work [12].

To this previously developed and validated model, we applied the newly developed computational strategies described below. In one of our analyses, we simulated a specific pathological-scarring scenario by modifying the values of two parameters in the default parameter set: we decreased by twofold the fibroblast apoptosis rate and increased by 1.5-fold the rate of collagen production by fibroblasts. We chose to modify these specific parameters because we had previously identified the biological processes represented by them as likely mechanistic factors driving pathological scarring in wounds [12].

### Generation of 120,000 new wound-healing simulations

The rates of biological processes involved in wound healing are expected to naturally vary among different mammalian species and individual animals, and also to vary due to differences in wound area, depth, and location. To reflect such natural variation in the molecular and cellular processes involved in wound healing, we simulated 120,000 distinct wound-healing scenarios, using modified model-parameter values. The corresponding 120,000 parameter sets were generated by randomly selecting the values for the 108 model parameters from intervals spanning the ranges between one-half and double the default parameter values. Our decision to sample the model parameter values from within a 0.5–2-fold range of their default values was based on the detected variability in the time courses of inflammatory/proliferative cells and cytokines in the wounds of wild-type C57BL/6J mice [12]. We carried out all computations in the software suite MATLAB R2015b (MathWorks, Natick, MA), and solved the model equations by using the MATLAB solver DDE23 with default tolerance levels.

### Classification of scarring outcomes in wound-healing simulations

For each of the 120,000 simulations, we calculated the fold changes in the model variables representing collagen and fibroblast concentrations with respect to their concentrations in the simulation with the default parameter values. The fold changes were calculated at the final simulation time point of 40 days after wounding, which we assumed to be representative of the time required for a HTS to develop (i.e., ~ 6 weeks [4]). From these fold changes, we classified the 120,000 simulations into three groups (Fig. 1a). Simulations for which both collagen and fibroblast fold changes did not exceed 1 were classified as “normal healing” (i.e., healing resulting in a non-pathological scar). Those for which the fold changes were simultaneously ≥ 5 and ≤ 10 were classified as “mild pathological scarring.” Finally, simulations for which the fold changes exceeded 10 were classified as “severe pathological scarring.” Our choice of the cutoff values 5 and 10 for classifying a wound-healing outcome as “mild” or “severe” pathological scarring was based on published reports of experimentally measured increases in collagen synthesis and deposition of up to fivefold in HTSs and up to 20-fold in keloids [14–16]. If the fold changes did not satisfy the fold-change cutoffs for any of the three classification groups (i.e., fold changes > 1 and < 5), the wound-healing scenarios represented by those simulations could not be clearly classified as normal healing or as pathological scarring. The results of such simulations were excluded from the analysis of protein concentration distributions (Fig. 1a). While this step resulted in a large group of simulations being excluded from the analysis, it reduced the ambiguity in prognostic biomarker identification by considering only the simulations that were most likely to represent normal-healing or pathological-scarring wounds.

### Analysis of protein concentration distributions in normal-healing and pathological-scarring simulations

We generated protein concentration distribution histograms using the MATLAB function HIST with 50 bins partitioning the interval between the minimal and maximal concentration values for a protein in a particular group of simulations (i.e., normal healing, mild pathological scarring, or severe pathological scarring). These histograms were visualized as concentration distribution curves (Figs. 3 and 4). The percentage of the simulations for each curve was calculated by dividing the number of simulations in which a given protein’s concentration fell within the concentration range of a particular bin by the total number of simulations in that group. For each protein in our model (excluding collagen), we analysed the area of overlap between its concentration distribution generated from the normal-healing simulations and the corresponding distributions generated from the mild and moderate pathological-scarring simulations. A small overlap area indicates that the protein is consistently elevated (or decreased) in pathological-scarring simulations relative to normal-healing simulations, and was therefore more likely to be associated with a pathological outcome than proteins with larger distribution overlap areas (Fig. 1a). Concentration distributions have previously been utilized for biomarker identification, extending the prevalent practice based on fold-change analysis of gene/protein expression [17–19]. To quantify the area of overlap between the concentration distributions, we calculated the Bhattacharyya coefficient as previously described [19]. The value of this coefficient varies between 0 and 1, which represent 0 and 100% overlap, respectively. Proteins whose concentration distributions were characterized by little overlap between the normal-healing and pathological-scarring simulations on day 40 (i.e., upon complete HTS development) were determined to be *diagnostic* biomarkers of pathological scarring. In contrast, proteins whose concentration distributions were characterized by little overlaps between the normal-healing and pathological-scarring simulations on days 7, 14, and 21 post-wounding were determined to be putative *prognostic* biomarkers. When experimental data for the model-identified diagnostic biomarkers were available in the literature [14, 20–24], we compared the protein fold changes in pathological scars in humans (i.e., hypertrophic scars and keloids) derived from the experimental data using with the corresponding fold changes calculated from the pathological-scarring simulation described in the first subsection of “Methods” section.

### Analysis of predictive accuracy using logistic regression

**Fig. 2:**
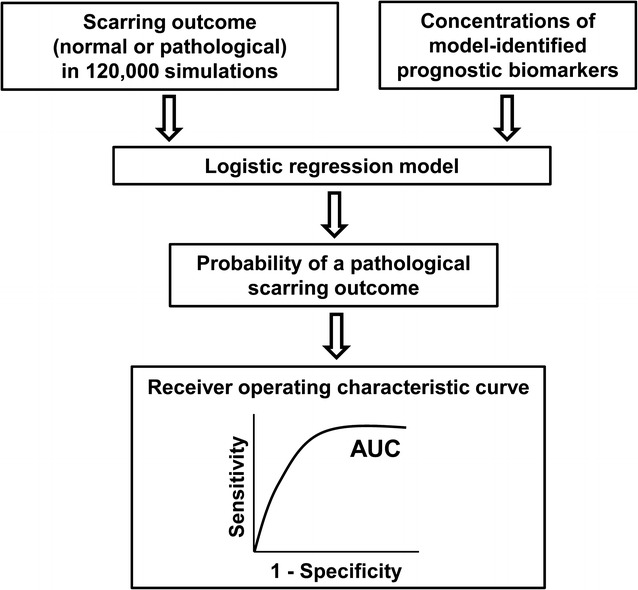
Logistic regression analysis. We provided the classification (i.e., “normal” or “pathological”) and the normalized concentrations of the model-identified prognostic biomarkers from the 120,000 simulations as inputs to logistic regression models. The models yielded logistic regression coefficients for each model-identified biomarker and the probability of a given simulation being “pathological” based on one, two, or three biomarkers as predictors. We used the probabilities resulting from the logistic regression models to derive the ROC curves (see Additional file 1 for further details)

We used logistic regression analysis to quantify the predictive accuracy of the prognostic biomarkers identified in our protein concentration distribution analysis (described above). For the logistic regression analysis, we used all of the 120,000 simulations and divided them into two groups: “normal healing” and “pathological scarring.” The simulations for which the fold change in the collagen level at the final simulation time point (i.e., day 40) exceeded 10 were classified as “pathological scarring,” and the remaining simulations were classified as “normal healing.” Next, the binary wound outcome (i.e., “normal” or “pathological”) of a simulation, along with the levels of the model-identified prognostic biomarkers at days 7, 14, and 21 in those simulations, were used as inputs to a logistic regression model (Fig. 2). The logistic regression model then yielded receiver operating characteristic (ROC) curves, which were used to quantify the predictive accuracy of the model-identified biomarkers (described in detail in the Additional file 1). Finally, using the logistic regression model that demonstrated the highest predictive accuracy, we performed a tenfold cross-validation analysis to estimate that model’s performance on an independent data set [25].

## Results

### Computational model identifies proteins elevated in pathological scars in humans

**Fig. 3:**
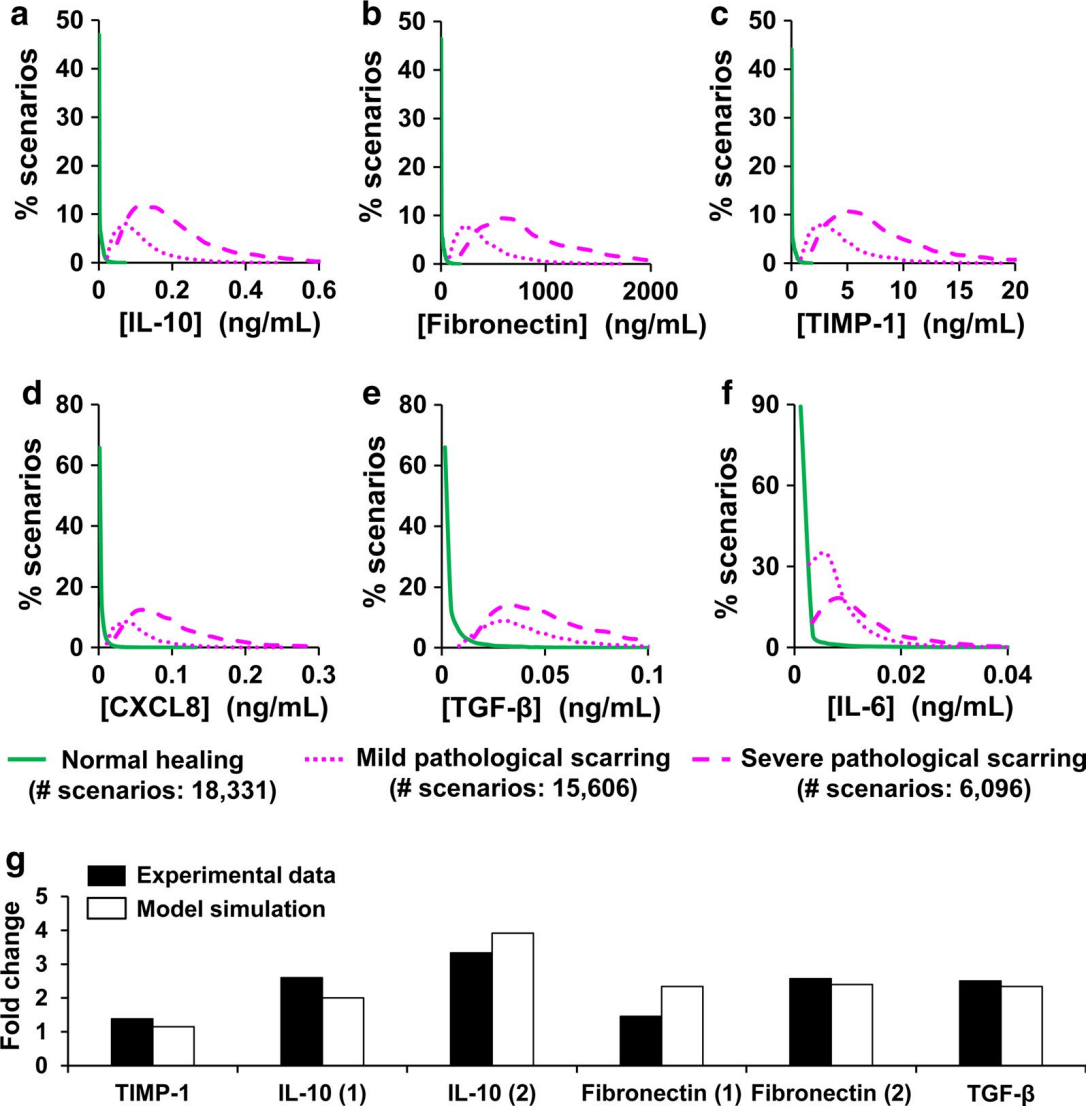
Diagnostic biomarkers of pathological scarring. Concentration distributions of **a** IL-10, **b** fibronectin, **c** TIMP-1, **d** CXCL8, **e** TGF-β, and **f** IL-6 in normal-healing simulations (solid green lines), mild pathological-scarring simulations (dotted pink lines), and severe pathological-scarring simulations (dashed pink lines) at the final simulated time point (i.e., day 40). Brackets (*x*-axis) designate concentrations. *y*-axis represents the percentage of simulations for each curve (described in “Methods” section). **g** Solid bars represent the fold changes in protein levels in human scar tissue calculated from published experimental data available in the literature. A fold change was calculated as the level of a protein measured in the material derived from pathological scar tissue divided by its corresponding level measured in the material derived from scar tissue under normal-healing conditions. The assay used to measure the level of a particular protein, as well as the time at which the measurement was performed, varied between different experimental studies. The data on TGF-β and TIMP-1 were taken from Refs. [21, 22], respectively. The data on IL-10 were taken from two separate studies: Ref. [20] [IL-10 (1)] and Ref. [23] [IL-10 (2)]. The data on fibronectin were also taken from two separate studies: Ref. [14] [Fibronectin (1)] and Ref. [24] [Fibronectin (2)]. Open bars represent the corresponding model-simulated fold change values. We have not found any published experimental data on the levels of CXCL8 and IL-6 in pathological scars

To identify biomarkers of HTSs, we first established criteria by which each of the wound-healing simulations could be classified as resulting in either normally healing or pathological scar formation. The pie chart in Fig. 1b shows the number of simulations falling into each group after the classification. To validate our biomarker identification methodology, we identified those proteins that were elevated (or decreased) in pathological-scarring simulations (both mild and severe) compared to normal healing simulations, and could therefore be regarded as diagnostic biomarkers for the condition. We found that six proteins—IL-10, TIMP-1, IL-6, TGF-β, chemokine CXCL8, and fibronectin—were characterized by relatively little overlap between their concentration distributions for normal-healing simulations and the corresponding distributions for mild pathological-scarring simulations and severe pathological-scarring simulations on day 40 (Fig. 3a–f).

To further assess the potential power of identifying diagnostic biomarkers using computational methods, we reviewed the existing literature to obtain the experimental measurements of TIMP-1, IL-10, fibronectin, and TGF-β in human pathological scars versus normally-healed wounds. The fold changes reported in human wounds were compared with the corresponding fold changes calculated from our simulations for normal-healing and pathological-scarring outcomes (Fig. 3g). The fold changes seen in our simulations showed agreement with the human experimental data. Differences in the experimentally obtained and model-simulated fold changes ranged from 6 to 60%), with 4 of the 6 factors having differences of less than 20% (Fig. 3g). The original data from published experimental studies using human scar tissue, including details about the protocols used for protein level measurement and the time-points at which the measurement were performed, are provided in Additional file 2.

### Putative prognostic biomarkers of pathological scarring

**Fig. 4:**
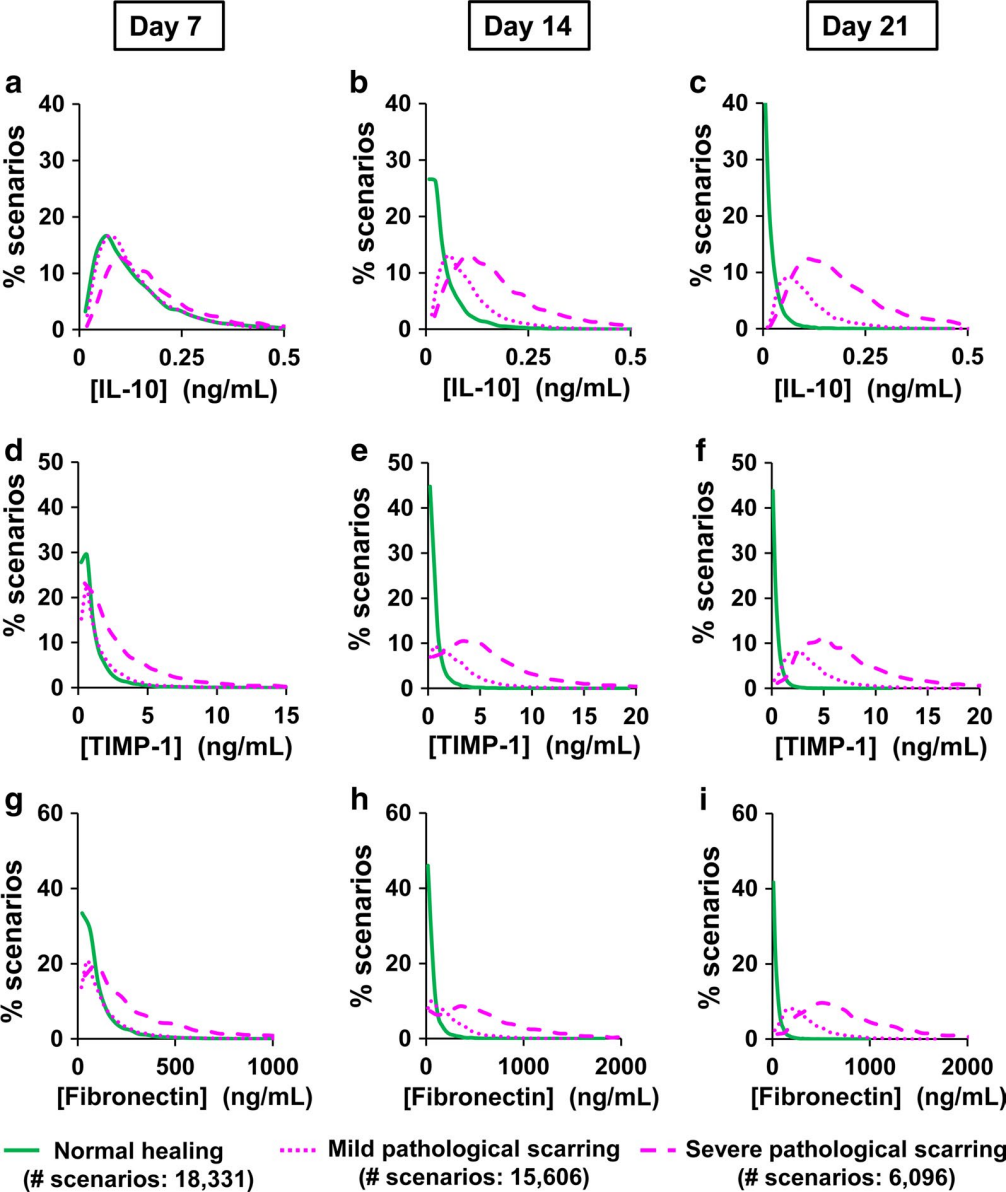
Putative prognostic biomarkers of pathological scarring. Concentration distributions of **a**–**c** IL-10, **d**–**f** TIMP-1, and **g**–**i** fibronectin at simulated times representing days 7, 14, and 21 post-wounding. Brackets (x-axis) designate concentrations. *y*-axis represents the percentage of simulations for each curve (described in “Methods” section). Solid green lines show the concentration distributions for the normal-healing simulations, dotted pink lines show the concentration distributions for the mild pathological-scarring simulations, and dashed pink lines show the concentration distributions for the severe pathological-scarring simulations

Having identified diagnostic proteins with high levels in late stage scars, we next sought to identify proteins that may serve as prognostic biomarkers of pathological scarring. In this analysis, we looked for proteins whose concentrations at early time points in the simulations (days 7, 14, and 21 post-wounding) were highly predictive of the scarring outcome (i.e., normal or pathological scarring) at the end of those simulations (i.e., day 40). We utilized the simulation data to identify proteins whose concentration distributions showed minimal overlap between simulations that resulted in normal-healing and those resulting in mild or severe pathological-scarring (Fig. 4). The analysis demonstrated that IL-10 (Fig. 4a–c), TIMP-1 (Fig. 4d–f), and fibronectin (Fig. 4g–i) were characterized by small overlap areas between normal-healing and pathological-scarring outcomes on days 14 and 21. This indicates that, as early as days 14 and 21, levels of IL-10, TIMP-1, and fibronectin may be predictive of a pathological-scarring outcome.

At the earliest of the three examined time points post-wounding (i.e., day 7), there was considerable overlap between the concentration distributions for the normal-healing and pathological-scarring simulations for IL-10, TIMP-1, and fibronectin (Fig. 4a, d, g). Thus, none of these proteins were predictive of the scarring outcome on day 7 post-wounding. On day 14, however, the area of overlap between the concentration distributions for each of these proteins in the normal-healing and pathological-scarring simulations was ~ 14–23% smaller than on day 7 (Fig. 4b, e, h). The overlap area for each of these proteins was the smallest on day 21 (~ 30–40% smaller than the corresponding overlap areas on day 7) (Fig. 4c, f, i). This suggests that IL-10, fibronectin, or TIMP-1 levels were most likely to successfully predict a pathological scarring outcome at or later than week 3 post-wounding.

Among IL-10, TIMP-1, and fibronectin, fibronectin was characterized by the smallest concentration distribution overlap between the normal-healing and pathological-scarring simulations on both days 14 and 21. On day 14, the fibronectin distribution overlap area was 14 and 5% smaller than the corresponding areas for IL-10 and TIMP-1, respectively; on day 21, the fibronectin distribution overlap area was 8 and 1.5% smaller than the corresponding areas for IL-10 and TIMP-1, respectively (Fig. 4h, i). The corresponding overlap areas for IL-10 and TIMP-1 were comparable at both 14 and 21 days. Thus, our distribution overlap analysis showed that IL-10, TIMP-1, and fibronectin are putative prognostic biomarkers of pathological scarring in wounds.

### Predictive accuracy of the putative prognostic biomarkers

**Fig. 5:**
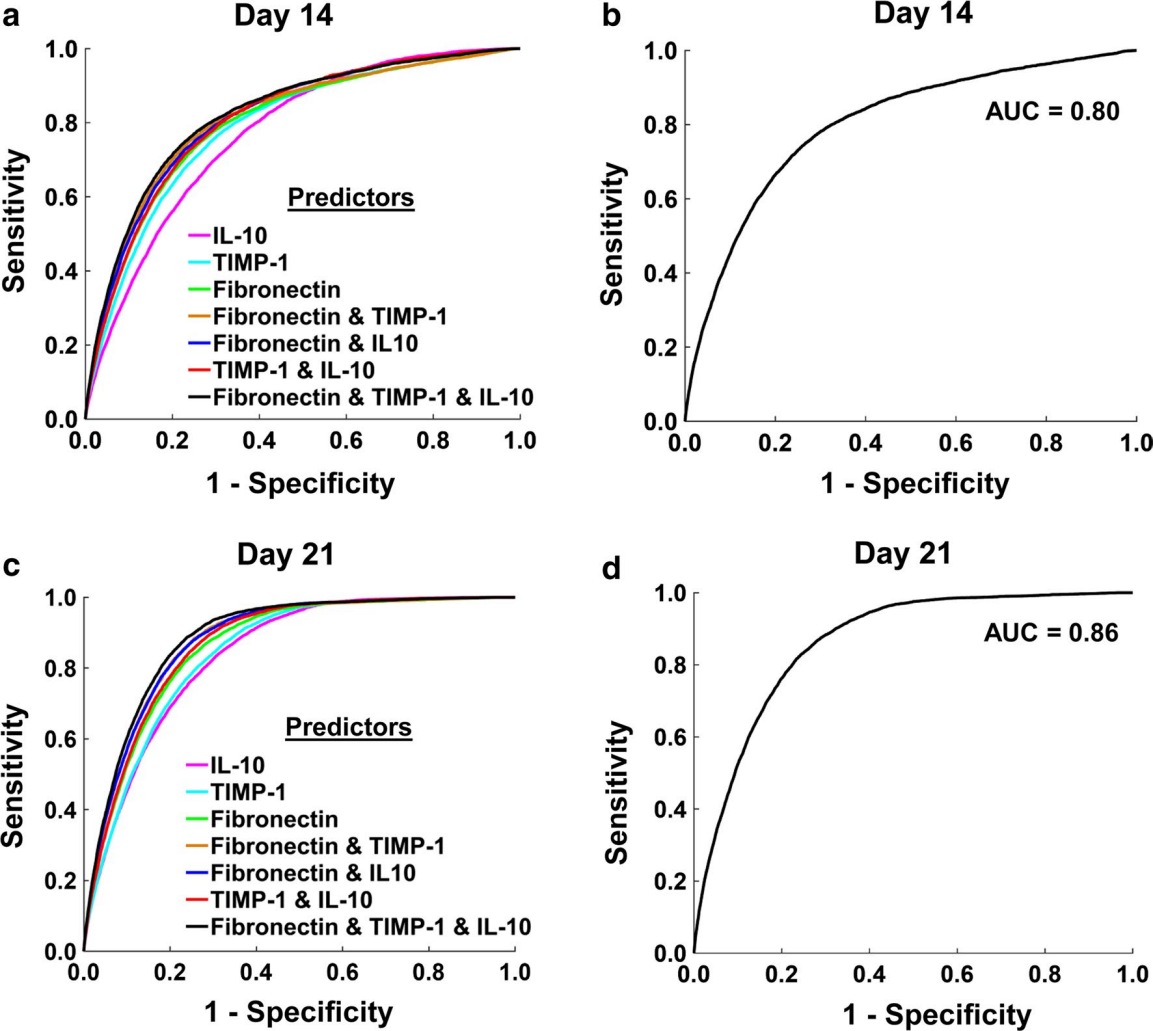
Receiver operating characteristic (ROC) curves. **a** ROC curves derived from logistic regression models, using day-14 concentrations of IL-10 alone (pink line, AUC: 0.77, 95% CI [0.767, 0.777]); TIMP-1 alone (cyan line, AUC: 0.79, 95% CI [0.784, 0.795]); fibronectin alone (green line, AUC: 0.80, 95% CI [0.794, 0.806]); TIMP-1 and IL-10 (red line, AUC: 0.81, 95% CI [0.806, 0.816]); fibronectin and TIMP-1 (brown line, AUC: 0.81, 95% CI [0.807, 0.818]), fibronectin and IL-10 (blue line, AUC: 0.82, 95% CI [0.811, 0.821]); and fibronectin, TIMP-1, and IL-10 (black line, AUC: 0.82, 95% CI [0.818, 0.829]). **b** ROC curve for tenfold cross validation performed by using day-14 concentrations of fibronectin, TIMP-1, and IL-10 as predictors (AUC: 0.80, 95% CI [0.792, 0.807]). **c** ROC curves derived from logistic regression models, using day-21 concentrations of IL-10 alone (pink line, AUC: 0.83, 95% CI [0.834, 0.842]); TIMP-1 alone (cyan line, AUC: 0.84, 95% CI [0.840, 0.848]); fibronectin alone (green line, AUC: 0.86, 95% CI [0.859, 0.866]); TIMP-1 and IL-10 (red line, AUC: 0.87, 95% CI [0.866, 0.873]); fibronectin and TIMP-1 (brown line, AUC: 0.87, 95% CI [0.875, 0.882]); fibronectin and IL-10 (blue line, AUC: 0.88, 95% CI [0.876, 0.883]); and fibronectin, TIMP-1, and IL-10 (black line, AUC: 0.89, 95% CI [0.884, 0.891]). **d** ROC for tenfold cross validation performed by using day-21 concentrations of fibronectin, TIMP-1, and IL-10 as predictors (AUC: 0.86, 95% CI [0.855, 0.870])

We next sought to quantitatively assess the predictive accuracy of the IL-10, TIMP-1, and fibronectin in two instances: (1) when only one protein was used to predict a pathological-scarring outcome or (2) when two or three protein levels were used together to predict the pathological-scarring outcome. We built 14 logistic regression models that used single protein concentrations, or combinations thereof, as predictors (seven regression models for day 14 post-wounding and another seven for day 21), and derived a ROC curve for each model (Fig. 5; see Additional file 1 for further details). The regression coefficients, odds ratios, and ROC AUCs for these logistic regression models are listed in Additional file 1: Table S1. Among the six models that used the concentration of only one protein as a predictor, the model utilizing the fibronectin concentration on day 21 as a predictor demonstrated the highest predictive accuracy (ROC AUC: 0.86) (Fig. 5c). Overall, the model that used the concentrations of IL-10, TIMP-1, and fibronectin on day 21 as predictors showed the highest accuracy (ROC AUC: 0.89) (Fig. 5c).

We performed DeLong’s test to determine if any one of the ROC AUCs was significantly greater than the rest. The ROC AUCs for the seven models that used the day-14 biomarker concentrations were not significantly different from one another (Fig. 5a). This result implies that using the day-14 concentrations of any of the three model-identified biomarker proteins as predictors individually or in combination with each other could predict a pathological-scarring outcome with similar success. In contrast, among the seven models that used the day-21 biomarker concentrations, the ROC AUC for the model that used IL-10, TIMP-1, and fibronectin as predictors was significantly greater than the ROC AUCs for models that used only one protein as a predictor (Fig. 5c). However, it did not differ significantly from the ROC AUCs of the models that used two proteins as predictors (Fig. 5c). To validate our assessment of predictive accuracy by using an independent data set (i.e., data that were not used to build the logistic regression models), we performed a tenfold cross-validation for the logistic regression models that used all three biomarkers (i.e., the IL-10, TIMP-1, and fibronectin concentrations) on either day 14 or 21 as predictors. The ROC AUCs derived from this analysis for days 14 and 21 equaled 0.80 and 0.86, respectively (Fig. 5b, d). Together, the data suggest that development of highly accurate predictive biomarkers for scar formation may involve a panel of markers rather than a single factor.

## Discussion

There is a pressing need for prognostic biomarkers to objectively predict whether a traumatic or surgical skin injury will result in excessive scarring [4, 26]. Few molecular markers have been validated and, to our knowledge, none are in active clinical use [10, 11, 27]. The identification of prognostic biomarkers for scar formation in humans is challenging, primarily because of the need for large scale prospective analysis of human samples. In the work described here, we demonstrate that computational modeling may be used to effectively predict prognostic markers. Markers identified by this method could provide an excellent starting point for validation in small-scale human studies. Our results suggested that the concentrations of IL-10, TIMP-1, and fibronectin in the wound at early time points may serve as prognostic biomarkers for HTSs. Logistic regression analysis showed that the levels of these proteins as early as 14 days post-wounding can indicate the risk of pathological scarring with an accuracy of ~ 80%, with the accuracy increasing to 86% when the markers are assessed at 21 days post-wounding. In particular, the accuracy of the regression model that used day-21 concentrations of all three proteins as predictors was significantly higher than that of the other regression models.

The findings described in this study demonstrate that our model can effectively identify diagnostic markers of HTSs. Diagnostic protein biomarkers (e.g., fibronectin and α-smooth muscle actin) can assist in differentiating pathological-scarring conditions of the skin (e.g., HTSs vs. keloids) [21, 28]. Moreover, they can provide insights into the molecular mechanisms of pathological scarring. In the clinic, pathological scars are typically characterized by visibly distinguishable features, such as raised skin, rigidity, redness, and morbidity [3–5, 9]. Therefore, their *diagnosis* typically does not rely on the use of protein biomarkers. In contrast, the *prognosis* of traumatic skin wounds would be greatly improved by the availability of clinically reliable prognostic biomarkers. The relative unpredictability of wound scarring outcomes strengthens the potential clinical usefulness of reliable prognostic biomarkers. While large-scale protein measurements are constrained by the availability of wound fluid or tissue, our computational modeling approaches can assist researchers in systematically screening and selecting putative biomarkers of pathological scarring [26]. Notably, computational analyses have been successfully used for such purposes for other wound pathologies. For example, the potential utility of IL-6 as an indicator of chronic inflammation, suggested by computational modeling efforts [13, 29], was independently confirmed in clinical and experimental studies [30, 31].

The identification and use of prognostic biomarkers of wound-healing outcomes is further complicated because the healing trajectory of a wound depends on the wound type and on specific wound characteristics, such as its area, depth, location, and closure rate. Despite this diversity, commonly occurring wound pathologies, such as HTSs and keloids, are typically characterized by excessive collagen synthesis and deposition [14, 15]. Consistently with this observation, we define “pathological scarring” as situations when wounds display excessive collagen levels compared to normal-healing wounds. HTSs have been reported in wounds of different types, including abrasions, lacerations, surgical incisions, and ‒ most frequently—burn wounds [3, 4, 15, 32, 33]. Thus, our model-identified protein biomarkers can be used to predict wound outcomes in all of these types of wounds. In practice, due to the constraints imposed by different wound characteristics (such as its location, area, and state of closure), our modeling results may be suitable only for clinical situations where reliable measurements of the model-identified proteins at weeks 2 and 3 post-injury are feasible.

Once prognostic markers for HTSs are established, an easy and effective sampling method will be required. Wound effluent is a promising source of candidate protein biomarkers predictive of pathological scarring [5, 34]. Indeed, some wound-effluent proteins have been linked to abnormal wound-healing conditions. For example, IL-6 levels are consistently elevated in dehisced traumatic wounds compared to wounds that healed normally [31, 35]. Moreover, the effluent from chronic ulcers is characterized by a high concentration ratio of MMP-9 to TIMP-1 [30, 36]. However, wound proteins have not been extensively investigated as biomarkers for pathological scarring in the skin. One possible reason for this is the limited availability of wound effluent (compared to serum) beyond early after injury, a situation that restricts opportunities for proteomic analysis. Although wound tissue samples might substitute for wound effluent, their accessibility during the early stages of wound healing is also restricted in terms of clinical considerations, quantity, patient condition, and patient consent [5]. Improvements in microsampling technologies may eventually overcome these obstacles.

**Fig. 6:**
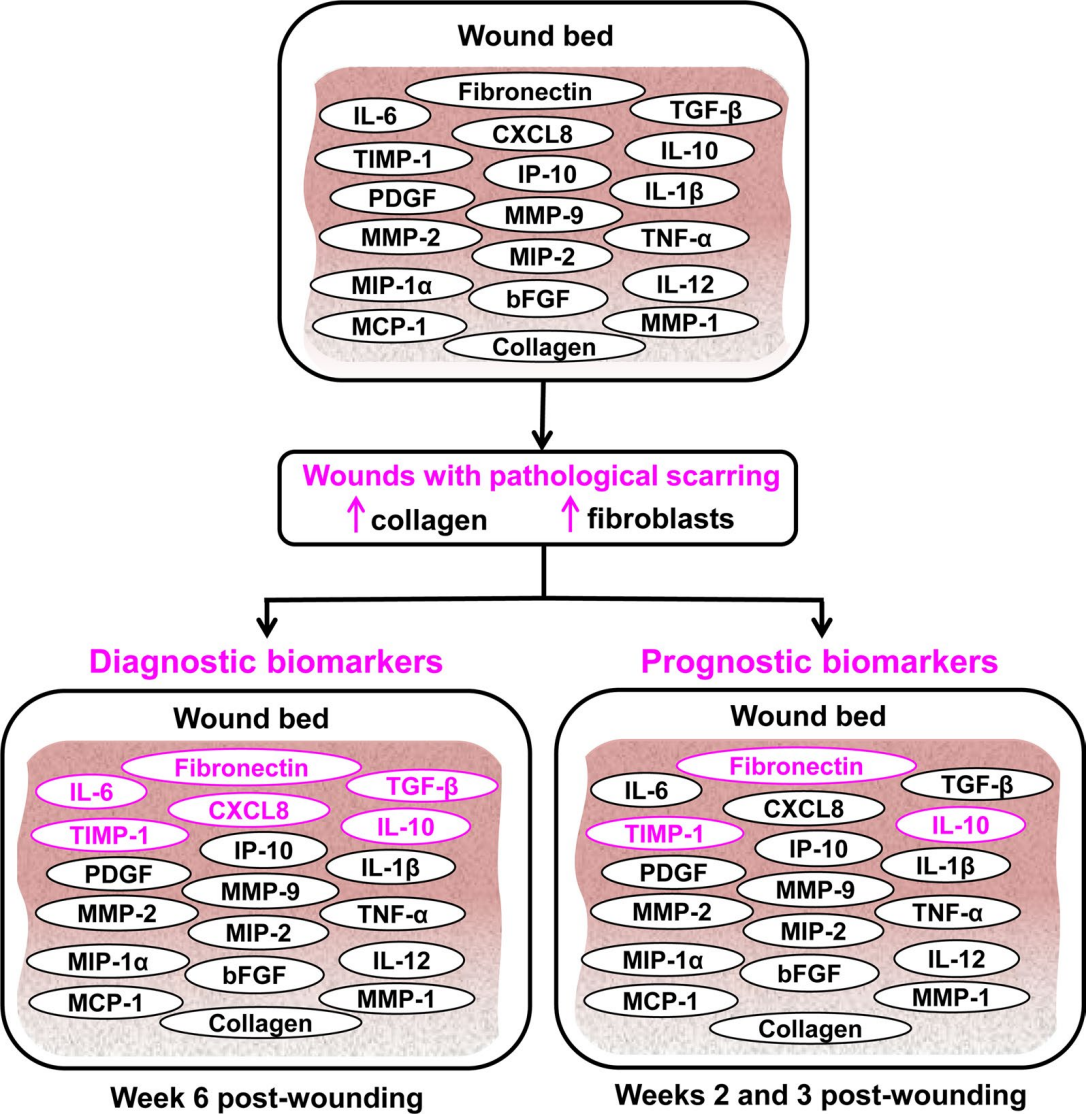
Summary of results. Among the 21 modeled proteins, six were shown to serve as diagnostic biomarkers of pathological scarring. Three modeled proteins were identified as putative prognostic biomarkers of pathological scarring with a reasonably high predictive accuracy (> 80%) on days 14 and 21 post-wounding

The putative protein biomarkers identified in this study (i.e., IL-10, TIMP-1, and fibronectin; Fig. 6) have been shown to exhibit diagnostic properties in fully developed HTSs or keloids in humans [14, 20, 22–24, 37–41]. Therefore, a similar correlation between the protein levels and pathological scarring could plausibly persist at earlier times during wound healing. This notion is supported by the fact that IL-10 is significantly elevated in the serum of burn injury patients who later develop HTSs and in the serum of animals and patients with fibrosis in the lung, intestine, and liver [20, 42]. IL-10 is the main anti-inflammatory cytokine involved in the later phase of inflammation and inhibits pro-inflammatory cytokine production [43, 44]. In addition to macrophages and T cells, IL-10 is produced by skin cells, such as keratinocytes and fibroblasts [45, 46]. The model-predicted capacity of IL-10 as a putative biomarker is in accord with this protein’s prominent role at the beginning of the proliferative phase. IL-10 can affect the expression of ECM effectors, such as MMP-1, MMP-8, and MMP-9 (both inhibitory and enhancing effects have previously been reported) [47], and enhance the production of TIMP-1 [43]. TIMP-1 is a glycoprotein that inhibits the action of collagen-degrading MMPs [22, 40]. High serum TIMP-1 levels characterize certain fibrotic diseases, such as liver cirrhosis, lung fibrosis, and skin fibrosis [39, 40]. Therefore, high levels of TIMP-1 in skin wounds are also likely to be predictive of pathological scarring. Among the three model-identified prognostic biomarkers, fibronectin demonstrated the highest predictive accuracy (Figs. 4 and 5). This is consistent with the biological function of fibronectin, which is the first ECM protein deposited during fibrogenesis [48]. Furthermore, fibronectin contributes to the regulation of collagen deposition by fibroblasts, conversion of fibroblasts to myofibroblasts, and promotion of wound contraction, all of which are essential for scarring [49].

The main limitations of this study arise due to the simplifying assumptions made during the computational model development and analysis. Nonetheless, our computational approach enabled systematic and efficient screening of proteins to identify putative biomarkers for future targeted testing. First, we used heuristic arguments to select the specific threshold values to classify our simulations as representing normal wound healing or pathological scarring. Those arguments reflected the fact that pathological scars typically have elevated collagen and fibroblast levels [4, 9, 14, 15, 33]. Second, our computational model represents 21 essential wound proteins, which form a subset of all the proteins present locally in the wound [50, 51]. Other wound proteins (e.g., angiogenic factors) not considered in our analysis may serve as biomarkers of pathological scarring. Although our model focuses on local factors, systemic factors—such as platelet availability, hormonal fluctuations, and systemic infections—may influence wound-healing outcomes. Because pathological scarring is frequently caused by local factors [52, 53], such systemic factors are not explicitly included in our model. Finally, we evaluated the predictive accuracy of the model-identified biomarkers at only three times (i.e., on days 7, 14, and 21). The choice of these times was based on the average time of discharge for surgical patients (~ 1–2 weeks) [54, 55].

## Conclusion

Our work illustrates how computational approaches can potentially increase the efficiency of experimental studies by generating testable hypotheses regarding putative prognostic biomarkers of pathological scarring in human wounds. Ultimately, these predictions need to be tested in human wounds to confirm the utility of these prognostic biomarkers in clinical settings. Computational models offer a non-invasive framework for evaluating current and emerging therapeutic strategies aimed to improve scarring outcome in wounds. Clinical validation of our model-predicted putative biomarkers could provide prognostic tools for objective, personalized clinical assessments of traumatic wounds.

## Additional files

**Additional file 1.** Description of the univariate and multivariate analysis of model-identified prognostic biomarkers of pathological scarring, list of modeled cell types and proteins, and Table S1 showing the logistic regression model coefficients, odds ratios, and AUCs.

**Additional file 2.** Original data from six published experimental studies that were used to derive the protein fold change values shown in Fig. 3g.

## Abbreviations

HTS: hypertrophic scar
ECM: extra cellular matrix
TGF-β: transforming growth factor-β
MMP: matrix metalloproteinase
IL: interleukin
TIMP-1: tissue inhibitor of matrix metalloproteinase-1
CXCL8: chemokine CXCL8
ROC AUC: area under receiver operating characteristic curve
