# Supplementary material for: Computational analysis identifies putative prognostic biomarkers of pathological scarring in skin wounds
Source: J Transl Med. 2018 Feb 20;16:32. doi: 10.1186/s12967-018-1406-x (PMC5819197; doi:10.1186/s12967-018-1406-x)
Supplement: Supplementary file 1 — Additional file 1. Description of the univariate and multivariate analysis of model-identified prognostic biomarkers of pathological scarring, list of modeled cell types and proteins, and Table S1 showing the logistic regression model coefficients, odds ratios, and AUCs. [file 12967_2018_1406_MOESM1_ESM.docx]

**Additional information**

**Computational analysis identifies putative prognostic biomarkers of pathological scarring in skin wounds**

by Sridevi Nagaraja, Lin Chen, Luisa A. DiPietro, Jaques Reifman, and Alexander Y. Mitrophanov

**ADDITIONAL METHODS INFORMATION**

For each putative prognostic biomarker identified using our computational kinetic model (see the main text), we developed a univariate logistic regression model with its concentration as a predictor. In addition, we developed a number of multivariate logistic regression models, wherein we used the concentrations of two or more identified putative prognostic biomarkers as predictors. We developed such multivariate logistic regression models for every possible combination of the identified putative biomarkers. First, we developed a set of logistic regression models that used biomarker concentrations on day 14, and then a set of analogous regression models that used the biomarker concentrations on day 21 post-wounding. All logistic regression models were developed using the MATLAB function FITGLM. For all the regression models, we used normalized protein concentration values and binary scarring outcomes (i.e., “normal” or “pathological”) from our 120,000 kinetic model simulations. Because the inference of regression coefficients generally depends on the scale of the inputs, and because the simulated concentration ranges for the proteins in the model spanned several orders of magnitude (0.01-2000 ng/mL), we normalized their concentration values by dividing them by two standard deviations as previously described.[^1^](#_ENREF_1)

Next, we derived receiver operating characteristic (ROC) curves for each logistic regression model. The ROC curve establishes the relationship between the sensitivity and specificity of a protein biomarker. The predictive accuracy of each protein biomarker was then defined as the area under the ROC curve (ROC AUC). Furthermore, we used DeLong’s test[^2^](#_ENREF_2) to determine whether the ROC AUCs were significantly different from each other (*P* ≤ 0.05 was considered significant). Finally, we performed a 10-fold cross-validation analysis, using the logistic regression model that produced the highest ROC AUC.[^3^](#_ENREF_3) This was carried out by using the MATLAB functions CROSSVALIND and PREDICT.

| **Table S1. Logistic regression models, coefficients, odds ratios, and AUCs. Brackets designate concentrations.** | | | |
| --- | --- | --- | --- |
| **Model** | **Coefficient estimates: X_i_ (mL·ng^-1^), Y_i_ (mL·ng^-1^),**  **a_i_ (dimensionless), and b_i_ (dimensionless)** | **Odds ratio (OR)** | **AUC** |
| **Day 14 concentrations** | | | |
| Predictor: [IL-10];  logit(y) = a_1_ + X_1_[IL-10] | a_1_ = −3.1  X_1_ = 1.2 | IL-10 OR = 3.3 | 0.77 |
| Predictor: [fibronectin];  logit(y) = a_2_ + X_2_[fibronectin] | a_2_ = −3.2  X_2_ = 1.4 | fibronectin OR = 4.0 | 0.80 |
| Predictor: [TIMP-1];  logit(y) = a_3_ + X_3_[TIMP-1] | a_3_ = –3.2  X_3_ = 1.3 | TIMP-1 OR = 3.7 | 0.79 |
| Predictors: [fibronectin] and [TIMP-1];  logit(y) = a_4_ + X_4_[fibronectin] + X_5_[TIMP-1] | a_4_ = –3.1  X_4_ = 1.0  X_5_ = 0.8 | fibronectin OR = 3.3  TIMP-1 OR = 2.5 | 0.81 |
| Predictors: [fibronectin] and [IL-10];  logit(y) = a_5_ + X_6_[fibronectin] + X_7_[IL-10] | a_5_ = –3.2  X_6_ = 1.1  X_7_ = 0.7 | fibronectin OR = 3.3  IL-10 OR = 2.5 | 0.82 |
| Predictors: [TIMP-1] and [IL-10];  logit(y) = a_6_ + X_8_[TIMP-1] + X_9_[IL-10] | a_6_ = −3.2  X_8_ = 1.0  X_9_ = 0.8 | TIMP-1 OR = 3.0  IL-10 OR = 2.8 | 0.81 |
| Predictors: [fibronectin], [TIMP-1], and [IL-10];  logit(y) = a_7_ + X_10_[fibronectin] + X_11_[TIMP-1] +  X_12_[IL-10] | a_7_ = −3.3  X_10_ = 0.8  X_11_ = 0.6  X_12_ = 0.6 | Fibronectin OR = 2.2  TIMP-1 OR = 2.1  IL-10 OR = 2.1 | 0.82 |
| **Day 21 concentrations** | | | |
| Predictor: [IL-10];  logit(y) = b_1_ + Y_1_[IL-10] | b_1_ = −3.2  Y_1_ = 1.5 | IL-10 OR = 4.5 | 0.84 |
| Predictor: [fibronectin];  logit(y) = b_2_ + Y_2_[fibronectin] | b_2_ = −3.3  Y_2_ = 1.7 | fibronectin OR = 5.5 | 0.86 |
| Predictor: [TIMP-1];  logit(y) = b_3_ + Y_3_[TIMP-1] | b_3_ = −3.2  Y_3_ = 1.5 | TIMP-1 OR = 4.5 | 0.84 |
| Predictors: [fibronectin] and [TIMP-1];  logit(y) = b_4_ + Y_4_[fibronectin] + Y_5_[TIMP-1] | b_4_ = −3.4  Y_4_ = 1.2  Y_5_ = 0.9 | fibronectin OR = 3.3  TIMP-1 OR = 2.5 | 0.88 |
| Predictors: [fibronectin] and [IL-10];  logit(y) = b_5_ + Y_6_[fibronectin] + Y_7_[IL-10] | b_5_ = −3.4  Y_6_ = 1.2  Y_7_ = 0.9 | fibronectin OR = 3.3  IL-10 = 2.5 | 0.88 |
| Predictors: [TIMP-1] and [IL-10];  logit(y) = b_6_ + Y_8_[TIMP-1] + Y_9_[IL-10] | b_6_ = −3.4  Y_8_ = 1.2  Y_9_ = 1.0 | TIMP-1OR = 3.0  IL-10 OR = 2.8 | 0.87 |
| Predictors: [fibronectin], [TIMP-1] and [IL-10];  logit(y) = b_7_ + Y_10_[fibronectin]+Y_11_[TIMP-1] +  Y_12_[IL-10] | b_7_ = −3.5  Y_10_ = 1.0  Y_11_ = 0.7  Y_12_ = 0.7 | fibronectin OR = 2.8  TIMP-1OR = 2.1  IL-10 OR = 2.1 | 0.89 |

**Computational model of wound healing: protein and cell-type variables**

| **Cell types** |  |
| --- | --- |
| Active neutrophils |  |
| Apoptotic neutrophils |  |
| Pro-inflammatory macrophages |  |
| Anti-inflammatory macrophages |  |
| Fibroblasts |  |
| Myofibroblasts |  |
| **Proteins Protein extended names** | |
| *CXCL*8 | Chemokine *CXCL*8 |
| *IL*12 | Interleukin-12 |
| *IL10* | Interleukin-10 |
| *FGF* | Fibroblast growth factor |
| *MMP1* | Matrix metalloproteinase-1 |
| *MCP*1 | Monocyte chemo attractant protein-1 |
| *TGFβ* | Transforming growth factor-β |
| *PDGF* | Platelet-derived growth factor |
| *IL1β* | Interleukin-1β |
| *IL6* | Interleukin-6 |
| *MIP*1*α* | Macrophage inflammatory protein-1α |
| *MIP*2 | Macrophage inflammatory protein-2 |
| *IP*10 | Interferon-γ-induced protein 10 |
| *TNFα* | Tumor necrosis factor-α |
| *Fibnec* | Fibronectin |
| *MMP9* | Matrix metalloproteinase-9 |
| *MMP2* | Matrix metalloproteinase-2 |
| *TIMP*1 | Tissue inhibitor of matrix metalloproteinase-1 |
| Tropocollagen  Collagen fibril |  |
| Collagen fiber |  |

**REFERENCES**

1 Gelman A. Scaling regression inputs by dividing by two standard deviations. *Stat Med* 2008; **27**: 2865-73.

2 DeLong ER, DeLong DM, Clarke-Pearson DL. Comparing the areas under two or more correlated receiver operating characteristic curves: a nonparametric approach. *Biometrics* 1988; **44**: 837-45.

3 Duda RO, Hart PE, Stork DG. *Pattern Classification (2nd Edition)*: Wiley-Interscience. 2000.
