# Supplementary material for: Computational analysis identifies putative prognostic biomarkers of pathological scarring in skin wounds
Source: J Transl Med. 2018 Feb 20;16:32. doi: 10.1186/s12967-018-1406-x (PMC5819197; doi:10.1186/s12967-018-1406-x)
Supplement: Supplementary file 2 — Additional file 2. Original data from six published experimental studies that were used to derive the protein fold change values shown in Fig. 3g. [file 12967_2018_1406_MOESM2_ESM.pdf]

Additional data information

TIMP-1

Simon F, Bergeron D, Larochelle S et al. Enhanced secretion of TIMP-1 by human hypertrophic scar keratinocytes could contribute to fibrosis. Burns 2012; 38: 421-7

Biological material: Human scar tissue

Fig.3

Method: ELISA assay of TIMP-1 with conditioned media from wound myofibroblasts (wmyo) and hypertrophic scar myofibroblasts (Hmyo) after 24 h

TIMP-1 (ng/ml)	Wmyo (N=6)	Hmyo (N=6)
Mean	28.117	40.307
STD	1.507	2.877
Ratio	1.434	

IL-10 (1)

Tredget EE, Yang L, Delehanty M et al. Polarized Th2 cytokine production in patients with hypertrophic scar following thermal injury. J Interferon Cytokine Res 2006; 26: 179-89

Biological material: Human scar tissue

Fig. 2

Method: ELISA assay of IL-10 with conditioned media from peripheral blood mononuclear cells from patients with and without hypertrophic scars after 1 month post-burn

IL-10 (pg/ml)	No hypertrophic scar (N=16)	Hypertrophic scar (N=16)
Mean	4.724	12.825
STD	2.801	2.251
Ratio	2.715	

IL-10 (2)

Magliacani G, Stella M, Castagnoli C et al. Post-burn pathological scar: clinical aspects and therapeutic approach. Ann Burns Fire Disasters 1997; 10: 105-9

Biological material: Human scar tissue

Fig. 4

Method: Detection of IL-10 in scar tissues of burn patients after 6 months

Total patients: 293

% IL-10 positive samples	Active hypertrophic scars (N= 99)	Normotrophic scars (N= 61)
	100.000	30.000
Ratio	3.333	

Fibronectin (1)

Ashcroft KJ, Syed F, Bayat A. Site-specific keloid fibroblasts alter the behaviour of normal skin and normal scar fibroblasts through paracrine signalling. PLOS One 2013; 8: e75600

Biological material: Human scar tissue

Fig. 8

Method: In cell western blotting post 10 days in culture

Fibronectin (relative protein expression)	Normal skin fibroblasts (N=4)	Keloid skin fibroblasts (N=5)

Mean	345.354	472.414
SEM	29.630	62.069
Ratio	1.368	

Fibronectin (2)

Babu M, Diegelmann R, Oliver N. Fibronectin is overproduced by keloid fibroblasts during abnormal wound healing. Mol Cell Biol 1989; 9: 1642-50

Biological material: Human scar tissue

Fig. 3

Method: Metabolic labeling and immunoprecipitation with affinity-purified anti fibronectin antibody

Fibronectin biosynthesis in fibroblasts (relative rates)	Normal tissue (N=2)	Keloid tissue (N=6)
Mean	1.050	2.710
STD	0.050	0.980
Ratio	2.581	

TGF-β1

Suarez E, Syed F, Alonso-Rasgado T *et al.* Identification of biomarkers involved in differential profiling of hypertrophic and keloid scars versus normal skin. *Arch Dermatol Res* 2015; 307: 115-33

Biological material: Human scar tissue

Fig.14

Method: In-cell western blotting

TGF-β1 (relative protein expression)	Normal skin fibroblasts (N=14)	Keloid fibroblasts (N=14)
Mean	0.917	2.400
SEM	0.372	1.073
Ratio	2.618	

STD: Standard deviation

SEM: Standard error of the mean
